# Montreal Veterinary Medical Association

**Published:** 1897-05

**Authors:** B. A. Sugden

**Affiliations:** Secretary-Treasurer


Secretary.

### MONTREAL VETERINARY MEDICAL ASSOCIATION.

A SPECIAL meeting of the Association was held in the Library of the Faculty of Comparative Medicine, March 25th, at 5 o'clock. Owing to the illness of the President, Dr. Baker, and his consequent absence, the chair was occupied by Prof. Adami. After the Acting Secretary had read the minutes of the preceding meeting, and of the corresponding meeting of last year, the chairman addressed those present, and in concluding his remarks extended his best wishes to the graduating class, and trusted that they would maintain the well-deserved reputation of their school.

The Acting Secretary then presented the annual report, which showed the Society to be in a sound financial condition, and the Librarian made a few remarks with regard to the condition of the library.

Dr. Adami then made his report upon the essays read before the Society during the past session, the awards being as follows: The first prize was won by Mr. J. C. Parker, his essay being entitled "Artificial Impregnation;" the second prize by Mr. B. A. Sugden, "Pneumonia;" and the third prize by R. G. Matthew, "Peritonitis." In Dr. Adami's opinion the best work was brought before the Society by case-reports, and he thought it would be wise if these were in the future taken into account in awarding prizes.

The diploma of honorary fellowship was then conferred upon the following gentlemen: Messrs. Burns, Connelly, Hilliard, Killam, Moore, Matthew, Newcombe, Parker, Stevenson, Sugden, and Thayer.

Dean McEachran then addressed the meeting, urging upon the graduating members the importance of keeping up their connections with societies and subscribing to magazines, stating how, during his recent examinations of professional men in connection with government inspection, he had especially noticed the capable and up-to-date manner in which those gentlemen who maintained these relations had handled their subjects. Alumni societies, he said, which existed in New York, British Columbia, etc., were branching out from being merely headquarters for friendly gatherings to include the study of scientific subjects, and it was only at the last meeting of the McGill Alumni Society of British Columbia that they had voted fifty dollars, to be equally divided as prizes among the five faculties of McGill University; also that the Massachusetts Alumni Society of the Faculty of Comparative Medicine of McGill had voted a prize especially for the examination of horses for soundness. In an able manner he pointed out the difficulties the profession had overcome in order to reach its present position; how already Great Britain, Canada, and the United States were recognizing its importance, and how, without being a prophet or a prophet's son, he could see the day close at hand when the people of these countries, who at present, owing to ignorance, were daily poisoning their families with tuberculosis and other contagious diseases through neglecting the proper care of their domestic animals, from which their food was derived, would demand that every pound of beef, pork, or milk should have the professional stamp upon it as being fit for consumption ere it was placed on the market. As regards horses, he showed how the general drudge of the past, oftentimes a cripple, was rapidly being replaced by electrical machines, in consequence of which the horse of the future will be a valuable animal and in most cases a family pet, necessitating for his medical attendant an educated gentleman, who, like his brother in human medicine, must be capable of understanding his patient's psychic qualities. In conclusion, he expressed his best wishes for the departing students and congratulated the Society upon the useful work it was doing.

After a short address from Dr. Parker a vote of thanks to Dr. Adami was proposed and carried unanimously, after which the meeting adjourned.

B. A. SUGDEN,
Secretary-Treasurer.

